# Supplementary material for: Monitoring Vitamin B12 in Women Treated with Metformin for Primary Prevention of Breast Cancer and Age-Related Chronic Diseases
Source: Nutrients. 2019 May 7;11(5):1020. doi: 10.3390/nu11051020 (PMC6567263; doi:10.3390/nu11051020)
Supplement: Supplementary file 1 [file nutrients-11-01020-s001.pdf]

**Table S1.** Baseline anthropometric parameters

| | n. | min | 25th centile | median | 75th centile | max | IQR |
|---|---|---|---|---|---|---|---|
| Age (yrs) | 165 | 46.6 | 53 | 57.8 | 63.3 | 73 | 10.3 |
| Height (cm) | 165 | 144 | 155 | 159 | 164 | 173 | 9 |
| Weight (kg) | 165 | 57.7 | 68 | 74.2 | 83.3 | 132.3 | 15.3 |
| Waist circumference | 165 | 85 | 87 | 93 | 99.5 | 133 | 12.5 |

IQR=interquartile range ($75^{th}$ centile-$25^{th}$ centile)

**Table S2.** Hematologic variables

| | time | n. | min | 25th centile | median | 75th centile | max | IQR |
|---|---|---|---|---|---|---|---|---|
| Serum creatinine (mg/dL) | baseline | 165 | 0.5 | 0.7 | 0.8 | 0.8 | 1.2 | 0.1 |
| | 3rd year | 163 | 0.5 | 0.7 | 0.7 | 0.8 | 1.2 | 0.2 |
| Hematocrit | baseline | 165 | 28.5 | 40.3 | 42.2 | 44.0 | 49.1 | 3.7 |
| | 3rd year | 164 | 34.0 | 40.2 | 41.8 | 43.5 | 46.5 | 3.3 |
| Erythrocytes (x$10^6$) | baseline | 165 | 4.1 | 4.6 | 4.8 | 5.0 | 7.9 | 0.4 |
| | 3rd year | 164 | 3.9 | 4.5 | 4.7 | 5.0 | 6.1 | 0.4 |
| Hemoglobin (g/dL) | baseline | 165 | 9.7 | 13.3 | 13.9 | 14.5 | 15.7 | 1.2 |
| | 3rd year | 164 | 10.1 | 13.2 | 13.8 | 14.5 | 15.4 | 1.3 |

IQR=interquartile range ($75^{th}$ centile-$25^{th}$ centile)
